# Integrative Gene Expression Profiling Reveals G6PD-Mediated Resistance to RNA-Directed Nucleoside Analogues in B-Cell Neoplasms

**DOI:** 10.1371/journal.pone.0041455

**Published:** 2012-07-27

**Authors:** Samuel K. McBrayer, Michael Yarrington, Jun Qian, Gang Feng, Mala Shanmugam, Varsha Gandhi, Nancy L. Krett, Steven T. Rosen

**Affiliations:** 1 Robert H. Lurie Comprehensive Cancer Center, Northwestern University, Chicago, Illinois, United States of America; 2 Division of Hematology and Oncology, Feinberg School of Medicine, Northwestern University, Chicago, Illinois, United States of America; 3 Feinberg School of Medicine, Northwestern University, Chicago, Illinois, United States of America; 4 Biomedical Informatics Center, Northwestern University, Chicago, Illinois, United States of America; 5 Department of Experimental Therapeutics, M.D. Anderson Cancer Center, University of Texas, Houston, Texas, United States of America; University of Navarra, Center for Applied Medical Research, Spain

## Abstract

The nucleoside analogues 8-amino-adenosine and 8-chloro-adenosine have been investigated in the context of B-lineage lymphoid malignancies by our laboratories due to the selective cytotoxicity they exhibit toward multiple myeloma (MM), chronic lymphocytic leukemia (CLL), and mantle cell lymphoma (MCL) cell lines and primary cells. Encouraging pharmacokinetic and pharmacodynamic properties of 8-chloro-adenosine being documented in an ongoing Phase I trial in CLL provide additional impetus for the study of these promising drugs. In order to foster a deeper understanding of the commonalities between their mechanisms of action and gain insight into specific patient cohorts positioned to achieve maximal benefit from treatment, we devised a novel two-tiered chemoinformatic screen to identify molecular determinants of responsiveness to these compounds. This screen entailed: 1) the elucidation of gene expression patterns highly associated with the anti-tumor activity of 8-chloro-adenosine in the NCI-60 cell line panel, 2) characterization of altered transcript abundances between paired MM and MCL cell lines exhibiting differential susceptibility to 8-amino-adenosine, and 3) integration of the resulting datasets. This approach generated a signature of seven unique genes including *G6PD* which encodes the rate-determining enzyme of the pentose phosphate pathway (PPP), glucose-6-phosphate dehydrogenase. Bioinformatic analysis of primary cell gene expression data demonstrated that G6PD is frequently overexpressed in MM and CLL, highlighting the potential clinical implications of this finding. Utilizing the paired sensitive and resistant MM and MCL cell lines as a model system, we go on to demonstrate through loss-of-function and gain-of-function studies that elevated G6PD expression is necessary to maintain resistance to 8-amino- and 8-chloro-adenosine but insufficient to induce *de novo* resistance in sensitive cells. Taken together, these results indicate that G6PD activity antagonizes the cytotoxicity of 8-substituted adenosine analogues and suggests that administration of these agents to patients with B-cell malignancies exhibiting normal levels of *G6PD* expression may be particularly efficacious.

## Introduction

The novel nucleoside analogues 8-amino-adenosine (8-NH_2_-Ado) and 8-chloro-adenosine (8-Cl-Ado) have undergone intensive preclinical development for cancer treatment by our laboratories due to the unique RNA-directed effects they elicit in tumor cells. These effects contrast the DNA-specific perturbations of other members of this drug class, such as cytarabine and gemcitabine. As a result, 8-NH_2_-Ado and 8-Cl-Ado show strong activity against indolent hematological malignancies characterized by intrinsically low rates of DNA replication and poor responsiveness to classical nucleoside analogues. Cellular conversion of these antimetabolites to their respective triphosphorylated forms is a prerequisite for induction of their pleiotropic activities leading to cell killing. This concept is supported by the observation that cells lacking adenosine kinase expression are completely resistant to 8-NH_2_-Ado or 8-Cl-Ado treatment [1], [2]. The mechanisms of action of these agents exhibit significant overlap due to their structural similarity; common activities include reduction of endogenous ATP pools and induction of bioenergetic stress as well as inhibition of RNA synthesis [1], [3]–[6]. Compromised ATP generation coupled with direct transcriptional incorporation of the analogues (resulting in chain termination) and disruption of polyadenylation leads to a profound and selective suppression of mRNA synthesis [7]. Through this mechanism, 8-NH_2_-Ado and 8-Cl-Ado exploit the reliance of cancer cells on continuous transcription of prosurvival genes encoding short half life proteins to maintain viability. For example, mRNA and protein quantities of the receptor tyrosine kinase c-Met fall rapidly in multiple myeloma cells exposed to 8-Cl-Ado [8] and Mcl-1 expression declines within hours of treatment initiation with either analogue in CLL cells [5], [9]. In addition to these shared properties, 8-NH_2_-Ado exhibits compound-specific attributes which may account for its increased potency in relation to 8-Cl-Ado. 8-NH_2_-Ado acutely suppresses glucose consumption in multiple myeloma cells [10] (which is associated with intracellular sequestration of GLUT4 and activation of autophagy) and elicits dephosphorylation and inactivation of Akt, mTOR and Erk kinases in a cancer-specific manner [3]. 8-NH_2_-Ado has also been shown to elicit cytotoxicity in a p53-independent manner (personal communication, Dr. Jill Bargonetti, Hunter College). The promising activity profiles of these compounds established through preclinical testing in MM, CLL, and MCL disease models have led to the initiation of an ongoing Phase I clinical trial of 8-Cl-Ado in CLL. MCL has the worst prognosis among all non-Hodgkin lymphomas [11] and all three malignancies are currently regarded as incurable [12]–[14]; therefore, there exists a great need for the development of new therapeutics which are effective against these diseases. Accumulation of high micromolar to low millimolar levels of 8-Cl-ATP in peripheral blood mononuclear cells (PBMC) following administration of 8-Cl-Ado to mice and rats [15] provides *in vivo* evidence supporting the auspicious clinical prospects of 8-substituted adenosine analogues to treat lymphoid neoplasms.

Despite our substantial efforts aimed at elucidating the mechanisms of action, our current understanding of the key determinants of sensitivity to 8-NH_2_-Ado and 8-Cl-Ado in MM, CLL, and MCL cells remains limited. Gaining deeper insight into this issue is crucial in order to facilitate the development of rational combinatorial therapeutic regimens, guide the design of future clinical trials, and illuminate cellular processes and molecular networks capable of circumventing the hallmark anticancer activities of these compounds. Our studies have not revealed a significant correlation between the absolute intracellular concentration of triphosphorylated analogue and responsiveness to these agents in cell lines exhibiting heterogeneous outcomes following treatment. For example, the resistant U266 and sensitive MM.1S myeloma cell lines exhibit equivalent peak intracellular 8-NH_2_-ATP concentrations of 3 mM following treatment with 3 µM 8-NH_2_-Ado [10]. Furthermore, 3 µM 8-NH_2_-Ado treatment of the sensitive JeKo MCL cell line results in intracellular accumulation of 3 mM 8-NH_2_-ATP and 50% cell death after 24 hours whereas 30 µM 8-NH_2_-Ado treatment of the resistant Granta 519 line over the same duration causes accrual of 17 mM 8-NH_2_-ATP but elicits only 25% cell death [3]. Based on these discrepancies, we hypothesized that specific patterns of gene expression may underlie intrinsic sensitivity or resistance to 8-substituted adenosine analogues. Therefore, we conceived a novel two-tiered, integrative chemoinformatic screen to uncover gene expression patterns which are: 1) associated with sensitivity or resistance to both compounds, 2) selected on a highly stringent basis with robust statistical significance, and 3) specifically applicable in the context of hematological malignancies of B-cell origin. Our screen identified seven unique genes meeting these criteria, one of which was *G6PD*. This gene encodes monomeric protein constituents of the metabolic enzyme glucose-6-phosphate dehydrogenase which occupies the key rate-limiting step of the PPP [16]. This glucose-dependent pathway produces cellular reducing power in the form of NADPH as well as ribose-5-phosphate for *de novo* purine biogenesis. G6PD occupies the most proximal step in the pathway, converting hexokinase-derived glucose-6-phosphate into 6-phosphogluconolactone through the coupled reduction of NADP^+^ to NADPH. Interestingly, this enzyme has numerous documented ties to cancer biology owing in part to its critical role in cell growth. Ectopic G6PD expression has been shown to confer tumorigenic potential to NIH3T3 cells, highlighting an oncogenic effect of elevated PPP flux [17]. Furthermore, increased expression of G6PD has been documented in cancers of the lung, breast, colon, prostate, cervix, and endometrium [18]. Our data demonstrate a unique requirement for elevated G6PD activity in 8-Cl-Ado/8-NH_2_-Ado-resistant MM and MCL cell lines to maintain the refractory phenotype. Our findings add to the growing knowledge base underlying the association between G6PD and chemoresistance and bolster the rationale for development of G6PD-targeting therapeutics.

## Results

### Interrogation of the NCI-60 cell line panel for gene expression associations with 8-Cl-Ado activity

In order to generate a molecular signature of responsiveness to 8-NH_2_-Ado and 8-Cl-Ado and identify genes capable of modulating their activities, we first determined the 8-Cl-Ado GI_50_ value for each tumor cell line within the NCI-60 panel. This data was generated through the Rapid Access to Intervention Development (RAID) initiative offered through the Developmental Therapeutics Program at the National Cancer Institute. Next, using a publicly available microarray study of the NCI-60 panel we performed linear regression analysis comparing 8-Cl-Ado GI_50_ values with gene expression values derived from unique oligonucleotide probes across all 60 cell lines. This approach enabled the calculation of Pearson correlation coefficients for over 7,000 gene-drug combinations. A graphical representation of the association between LGALS1 expression (Affymetrix probe J04456_at) and 8-Cl-Ado GI_50_ concentrations is included as Figure S1. The top 50 probes and corresponding transcripts positively associated with resistance to 8-Cl-Ado are detailed in Table 1 and a comprehensive list of all significant (*P*<.05) correlations is included in Table S1. Likewise, the 50 probes associated most strongly with sensitivity to 8-Cl-Ado are provided in Table 2 and the full list available as Table S2. In sum, 551 unique transcripts were identified to meet the statistical cutoff and were included in subsequent analyses.

**Table 1:** Gene expression patterns positively correlated with 8-Cl-Ado resistance.

Rank	Probe Set ID	Gene Symbol	r	*P* value
**1**	J04456_at	LGALS1	0.541833	7.79E-06
**2**	D45917_s_at	TIMP3	0.540177	8.40E-06
**3**	U14394_at	TIMP3	0.5315	1.24E-05
**4**	M36341_at	ARF4	0.522613	1.84E-05
**5**	L14848_s_at	MICA	0.479438	0.000106
**6**	D12485_at	ENPP1	0.467591	0.000165
**7**	L19711_at	DAG1	0.431138	0.000584
**8**	X52599_at	NGF	0.427406	0.000659
**9**	M15059_at	FCER2	0.427116	0.000665
**10**	X53002_s_at	ITGB5	0.426866	0.000671
**11**	Z54367_s_at	PLEC	0.426377	0.000681
**12**	M55621_at	MGAT1	0.42595	0.000691
**13**	S67247_s_at	MYH10	0.423273	0.000753
**14**	Y08999_at	ARPC1A	0.422093	0.000782
**15**	D31762_at	TRAM2	0.417506	0.000904
**16**	X86809_at	PEA15	0.415101	0.000974
**17**	M14676_at	FYN	0.415045	0.000976
**18**	X83416_s_at	PRNP	0.412563	0.001054
**19**	M94345_at	CAPG	0.411344	0.001094
**20**	HG987-HT987_at	IGFBP7	0.407883	0.001217
**21**	Y00433_at	GPX1	0.405977	0.001289
**22**	L06419_at	PLOD1	0.402979	0.001411
**23**	M91670_at	UBE2S	0.399234	0.001578
**24**	J03040_at	SPARC	0.399167	0.001581
**25**	M36430_s_at	GNB1	0.396747	0.001699
**26**	U53204_at	PLEC	0.393282	0.00188
**27**	M24470_at	G6PD	0.393087	0.001891
**28**	M65085_at	FSHR	0.392808	0.001906
**29**	X93510_at	PDLIM4	0.39206	0.001948
**30**	X04828_at	GNAI2	0.387137	0.002245
**31**	M29277_s_at	MCAM	0.38412	0.002446
**32**	J04605_at	PEPD	0.383521	0.002488
**33**	X00351_f_at	ACTB	0.383294	0.002504
**34**	X12492_at	NFIC	0.382948	0.002528
**35**	X96719_at	CLEC2B	0.381127	0.002661
**36**	M13577_at	MBP	0.380867	0.002681
**37**	X68487_at	ADORA2B	0.37992	0.002753
**38**	X04412_at	GSN	0.379788	0.002763
**39**	M16279_at	CD99	0.379596	0.002778
**40**	M13194_at	ERCC1	0.379253	0.002805
**41**	U56244_at	TDRD9	0.37888	0.002834
**42**	HG2981-HT3125_s_at	CD44	0.377902	0.002912
**43**	D85433_at	COMMD1	0.377538	0.002942
**44**	U09937_rna1_s_at	PLAUR	0.376902	0.002994
**45**	Z19554_s_at	VIM	0.376885	0.002995
**46**	M28882_s_at	MCAM	0.375844	0.003083
**47**	U13220_at	FOXF2	0.371321	0.00349
**48**	HG2743-HT2846_s_at	CALD1	0.369192	0.003697
**49**	HG3395-HT3573_s_at	DNAJB2	0.369033	0.003713
**50**	X56494_at	PKM2	0.368555	0.003761

**Table 2:** Gene expression patterns negatively correlated with 8-Cl-Ado resistance.

Rank	Probe Set ID	Gene Symbol	r	*P* value
**1**	U12595_at	TRAP1	−0.49975	4.80E-05
**2**	Z30425_at	NR1I3	−0.48648	8.11E-05
**3**	X64707_at	RPL13	−0.47666	0.000118
**4**	X04347_s_at	HNRNPA1	−0.46184	0.000204
**5**	D63874_at	HMGB1	−0.45152	0.000293
**6**	U70439_s_at	ANP32B	−0.44649	0.000349
**7**	AC002115_cds4_at	UPK1A	−0.43898	0.00045
**8**	M77140_at	GAL	−0.43825	0.000461
**9**	D50913_at	PMPCA	−0.42605	0.000689
**10**	D86964_at	DOCK2	−0.42394	0.000737
**11**	K02574_at	PNP	−0.42332	0.000752
**12**	Z14093_at	BCKDHA	−0.42137	0.0008
**13**	X69391_at	RPL6	−0.42075	0.000816
**14**	M36803_at	HPX	−0.41861	0.000873
**15**	D31763_at	ZNF33A	−0.41778	0.000896
**16**	HG4679-HT5104_at	RET	−0.41667	0.000928
**17**	X62534_s_at	HMGB2	−0.41589	0.000951
**18**	Z11850_at	GHR	−0.41516	0.000972
**19**	HG2365-HT2461_at	GAPDH	−0.41021	0.001133
**20**	X75091_s_at	SET	−0.41005	0.001139
**21**	M94046_at	MAZ	−0.40677	0.001259
**22**	HT3600_s_at	GCH1	−0.40592	0.001291
**23**	M84526_at	CFD	−0.40421	0.00136
**24**	X15940_at	RPL31	−0.4021	0.001449
**25**	L09708_at	C2	−0.39911	0.001584
**26**	D80004_at	KIAA0182	−0.39689	0.001691
**27**	M31523_at	TCF3	−0.39566	0.001754
**28**	U08471_at	FOLR3	−0.39234	0.001932
**29**	X73460_at	RPL3	−0.39144	0.001983
**30**	AF009674_at	AXIN1	−0.38879	0.002141
**31**	M55409_s_at	EEF1G	−0.38631	0.002298
**32**	L00058_at	MYC	−0.38337	0.002498
**33**	X80909_at	NACA	−0.38076	0.002689
**34**	M61827_rna1_s_at	SPN	−0.37897	0.002826
**35**	U12707_s_at	WAS	−0.37783	0.002918
**36**	X52056_at	SPI1	−0.3769	0.002994
**37**	X55733_at	EIF4B	−0.37578	0.003088
**38**	D80009_at	BMS1	−0.37494	0.003161
**39**	X55715_at	RPS3	−0.37273	0.003358
**40**	AC000061_cds3_at	CFTR	−0.37139	0.003483
**41**	X59303_s_at	VARS	−0.37021	0.003596
**42**	D87457_at	ELMO1	−0.36926	0.003691
**43**	X78136_at	PCBP2	−0.36801	0.003817
**44**	S82075_at	REV3L	−0.36684	0.003939
**45**	X67098_at	ENOSF1	−0.36678	0.003946
**46**	L28010_at	HNRNPF	−0.3656	0.004072
**47**	D86331_s_at	MMP15	−0.36408	0.004241
**48**	D21262_at	NOLC1	−0.36294	0.004371
**49**	L07540_at	RFC5	−0.36126	0.00457
**50**	Z47043_at	STARD9	−0.35925	0.004817

### Development of a gene expression signature predictive of responsiveness to 8-Cl-Ado and 8-NH_2_-Ado

**Figure 1:**
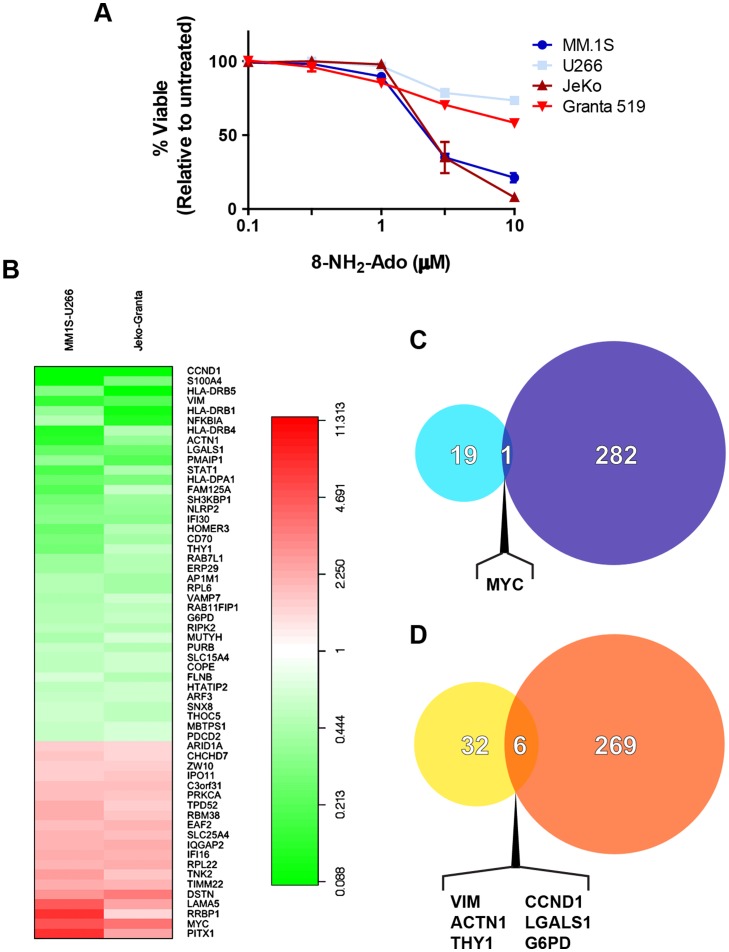
Transcriptomic microarray datasets independently identify seven mRNAs highly associated with responsiveness to 8-substituted adenosine analogues. (A) Paired sensitive and resistant MCL (JeKo and Granta 519, respectively) and MM (MM.1S and U266, respectively) cell lines were treated with a range of concentrations of 8-amino-adenosine for 24 hours before flow cytometric quantification of cell viability was performed using both DAPI and AnnexinV-FITC staining. Data are means ± SEM (*n* = 3). (B) Cell lines from part (A) under basal conditions were subjected to transcriptomic microarray analysis. Expression of genes significantly under- and over-expressed by at least a 1.5 fold change in sensitive cell lines relative to resistant paired cell lines are depicted and fold changes represented in the heatmap. (C) Integration of the list of genes highly expressed in sensitive cell lines in part (B) with genes highly expressed in sensitive constituent cell lines of the NCI-60 panel (Table 2 and Table S2) reveals overlap of one gene. (D) Integration of the list of genes highly expressed in resistant cell lines in part (B) with genes highly expressed in resistant constituent cell lines of the NCI-60 panel (Table 1 and Table S1) reveals overlap of six genes.

Next, we wished to refine the hits generated by this broad screening approach and select for transcripts which are: 1) particularly relevant in the context of B-cell malignancies and 2) predictive of responsiveness to activities common to both 8-NH_2_-Ado and 8-Cl-Ado. To accomplish this, we performed a small-scale gene expression profiling study to generate a second gene signature of responsiveness and integrated this dataset with that derived from the NCI-60 screen. In this small-scale study, two pairs of multiple myeloma and mantle cell lymphoma cell lines exhibiting dramatically differential sensitivity to 8-NH_2_-Ado were subjected to transcriptomic microarray analysis under basal conditions. As illustrated in Figure 1A, the sensitive MM.1S and JeKo cell lines exhibit LC_50_ concentrations of less than 3 µM whereas cell death in the resistant cell lines U266 and Granta 519 does not reach 50% with concentrations of 8-NH_2_-Ado ranging up to 10 µM. Importantly, these four cell lines also display differential sensitivity to 8-Cl-Ado [4], [19], indicating that intrinsic resistance mechanisms to common activities of both analogues are at play only in the U266 and Granta 519 cell lines. RNA isolated from each of the four cell lines was labeled and hybridized to Illumina Human-HT12 BeadChip microarrays. After signal intensity measurement and data processing were performed, gene expression patterns in sensitive cell lines deviating greater than 1.5 fold in a directionally-congruent manner from that in the corresponding resistant cell line were selected as markers of responsiveness to 8-NH_2_-Ado. Applying these criteria to the dataset yielded 20 genes negatively correlated with 8-NH_2_-Ado resistance and 38 genes exhibiting a positive correlation. This gene signature is depicted by the heatmap in Figure 1B in which red/green color intensity corresponds to the ratio of gene expression in the sensitive cell line relative to that in the resistant cell line. Finally, integration of the two gene signatures resulted in the identification of one overlapping gene associated with 8-NH_2_-Ado/8-Cl-Ado sensitivity and six overlapping genes associated with resistance (Figure 1C and D). Validation of the differential expression of *MYC, VIM*, *ACTN1*, *THY1*, *CCND1*, and *LGALS1* genes by real-time RT-PCR is included in Figure S2. The limited commonalities between the two datasets may reflect the importance of unique aspects of the mechanism of action of 8-NH_2_-Ado and/or the substantial differences in intrinsic gene expression patterns between the solid tumor-dominated NCI-60 panel and the MM/MCL cell lines represented in the secondary screen.

This highly stringent chemoinformatic approach revealed a surprising association between high expression of the classical oncogene *MYC* and sensitivity to these compounds. Given the intractability of developing pharmacological inhibitors of transcription factors and the widespread role c-myc plays in malignant transformation, our data indicate that the intrinsic properties of 8-NH_2_-Ado and 8-Cl-Ado may render these compounds particularly effective in treating tumors exhibiting c-myc deregulation, such as MM [13]. Considering the extremely short half-lives of c-myc mRNA and protein [20], transcriptional inhibition induced by these analogues may be expected to more potently suppress c-myc expression in relation to other genes, thereby offering an alternative therapeutic approach to targeting this oncogene in cancer. Additionally, we identified six genes associated with resistance to 8-NH_2_-Ado/8-Cl-Ado: *VIM*, *ACTN1*, *THY1*, *CCND1*, *LGALS1*, and *G6PD*. Of these six genes, both *VIM* and *ACTN1* encode cytoskeletal proteins (vimentin and alpha-actinin 1, respectively). Interestingly, the intermediate filament protein vimentin has been shown to functionally promote epithelial-to-mesenchymal transition (EMT) through stimulation of Axl expression [21] while upregulation of alpha-actinin 1 is a shared outcome of activation of the EMT regulators snail, slug, and E47 [22]. These data suggest that adoption of a mesenchymal phenotype may induce resistance to 8-substituted adenosine analogues, although the implications of these findings are difficult to decipher in the context of hematological malignancies. The *THY1* gene encodes the cell surface protein CD90 which has been demonstrated to be a marker for cancer stem cell populations in gastric and liver tumors [23], [24]. However, acute myelogenous leukemia stem cells exhibit reduced expression of this marker [25], thus clouding interpretation of the association of CD90 expression with a stem-like phenotype and drug resistance across diverse cancer types. The appearance of cyclin D1 (*CCND1*) is surprising given the fact that the t(11;14)(q13;q32) translocation (resulting in juxtaposition of IgH enhancer elements with the *CCND1* gene and cyclin D1 overexpression) is the genetic hallmark of mantle cell lymphoma [26] and is characteristic of both JeKo and Granta 519 MCL cell lines [27]. Further examination of cyclin D1 expression by real-time RT-PCR (Figure S2) confirms that U266 cells express approximately 90-fold greater levels of cyclin D1 in comparison to the MM.1S cell line. This result is expected given the fact that U266 cells harbor the t(11;14) translocation similar to the MCL lines whereas MM.1S cells lack this genetic lesion and are characterized by the chromosomal translocation t(14;16) targeting the c-Maf locus [28]. Analysis of cyclin D1 transcript abundance in the JeKo and Granta 519 MCL cell lines with three distinct primer/probe sets revealed that Granta 519 cells express between 2–2.5 fold greater cyclin D1 mRNA quantities in relation to JeKo cells. This difference is much smaller than that reflected in the microarray analysis, indicating that the microarray gene expression datasets may overstate the differences in cyclin D1 expression between these two lines. Immunoblot analyses of cyclin D1 protein expression (Figure S2) verify the dramatic difference in expression between the MM.1S and U266 cell lines but show virtually no difference in cyclin D1 expression between the JeKo and Granta 519 cell lines. This result indicates that JeKo cells likely exhibit more efficient cyclin D1 mRNA translation and/or diminished rates of cyclin D1 protein degradation such that the steady-state cyclin D1 protein levels in these two cell lines do not reflect the differences noted at the transcript level. In all microarray-based transcriptomic gene expression studies, incongruence between mRNA and protein expression levels of a given gene can result in the manifestation of false positive and false negative errors, thus necessitating caution in deriving conclusions from data pertaining only to mRNA abundance. Taken together, these results suggest that the t(11;14) genotype and high *CCND1* expression are associated with resistance to 8-NH_2_-Ado/8-Cl-Ado in the context of myeloma while the discrepancies between cyclin D1 transcript and protein abundances noted above suggest that this association is not meaningful in the context of mantle cell lymphoma. Interestingly, a novel anti-apoptotic role for cyclin D1 was recently reported in the literature relating to the ability of this protein to sequester BAX in the cytosol and prevent the initiation of apoptosis in response to Bcl-2 inhibition [29]. Given the ability of 8-NH_2_-Ado to interfere with Mcl-1expression [9] (the primary anti-apoptotic Bcl-2 family member expressed in myeloma), this cyclin D1-BAX interaction represents a potential mechanism accounting for the association between cyclin D1 overexpression and 8-NH_2_-Ado resistance in myeloma cells. In the case of *LGALS1*, a recent report characterized the induction of resistance to a variety of anticancer drugs following ectopic expression of galectin-3 in chronic myelogenous leukemia cells [30]. Our observation that galectin-1 (*LGALS1*) expression is predictive of 8-NH_2_-Ado/8-Cl-Ado responsiveness lends additional support to the previously unappreciated association of this gene family with chemoresistance. The gene derived from this signature that we regarded as the most compelling, however, was *G6PD*, which produces the enzyme glucose-6-phosphate dehydrogenase. Our interest in G6PD stemmed from knowledge that this protein functions in a glucose-dependent metabolic pathway and that sublethal glucose deprivation is sufficient to sensitize U266 cells to the actions of 8-NH_2_-Ado [10]. We hypothesized that heightened PPP flux may underlie instrinsic resistance to 8-NH_2_-Ado and 8-Cl-Ado and represent the fundamental cellular mechanism linking glucose metabolism with the activity of these agents.

### G6PD overexpression is observed in primary MM and CLL samples

**Figure 2:**
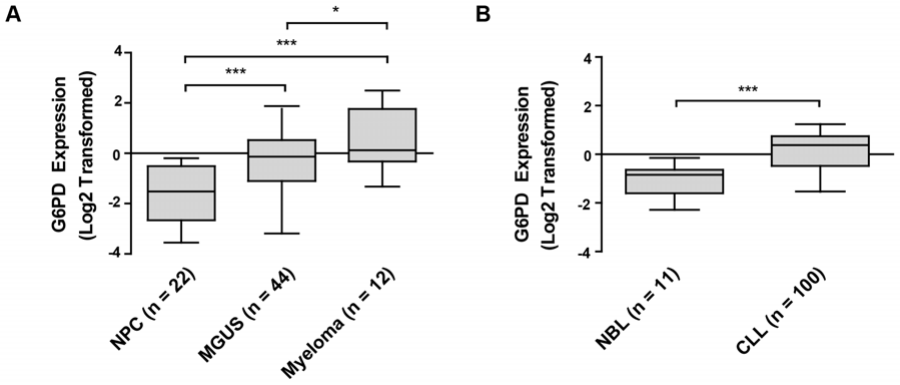
Increased G6PD expression is observed in MM and CLL patient samples. (A) G6PD expression was interrogated in clinical myeloma samples through querying the dataset ‘Zhan Myeloma 3’ for G6PD probe set ‘202275_at’ in the Oncomine™ database. MM samples exhibit a 4.745 fold increase in G6PD expression relative to normal plasma cells (NPC). MGUS: Monoclonal Gammopathy of Undetermined Significance. According to the authors Zhan et al [31], the diagnosis of MGUS included the following criteria: levels of monoclonal protein ≤30 g/L, plasma cell bone marrow infiltration <10%, and no incidence of related organ or tissue impairment. (B) G6PD expression was interrogated in clinical CLL samples through querying the dataset ‘Haslinger Leukemia’ for G6PD probe set ‘38043_at’ in the Oncomine™ database. CLL samples exhibit a 2.319 fold increase in G6PD expression relative to normal B lymphocytes (NBL). Data in parts (A) and (B) are log_2_ transformed and median-centered. Boxes indicate 95 and 5 percentiles and median expression values. Whiskers represent minimum and maximum expression values. * *P*<.05 ** *P*<.01 *** *P*<.005.

First, we endeavored to establish whether G6PD overexpression was clinically relevant to the pathogenesis of MM, MCL and CLL. Querying the Oncomine™ database for comparisons between normal lymphocytes and MM and CLL patient samples revealed strong evidence for G6PD overexpression in these malignancies. Of two studies meeting the search criteria for myeloma, both showed statistically significant G6PD overexpression in MM samples. Figure 2A (extracted from the dataset ‘Zhan Myeloma 3’) demonstrates a progressive increase in G6PD transcript abundance during transformation from MGUS to myeloma and significant overexpression in these cell types relative to normal plasma cells [31]. Specifically, MM samples exhibit a 4.745 fold increase in G6PD expression relative to normal plasma cells. The other dataset, ‘Zhan Myeloma’, demonstrated a 1.216 fold increase in G6PD expression in MM samples compared to normal plasma cells. Extrapolation of our findings in the MM.1S and U266 myeloma cell lines would suggest that there may be a select cohort of MM patients which display G6PD upregulation, thus causing this variance in the estimate of G6PD overexpression in clinical samples. Similar findings were also derived from studies in CLL in which two out of four applicable datasets showed statistically significant G6PD overexpression in CLL samples relative to normal B lymphocytes. The results from the dataset ‘Haslinger Leukemia’ [32] are included as Figure 2B and depict a 2.319 fold increase in G6PD expression. A fold change of 1.222 was reported in the dataset ‘Haferlach Leukemia’ but this may be an underestimate considering the nonmalignant control in this study is PBMC. Importantly, the two studies which did not corroborate these results exhibited features which confounded analysis; one study included only three CLL samples while the other utilized B lymphocytes procured during various stages of activation as controls rather than resting B cells. Unfortunately, no satisfactory datasets were identified in searches of the Oncomine™ and NCBI GEO databases to perform a similar analysis for mantle cell lymphoma. These data demonstrate that G6PD overexpression is widely observable in MM and CLL clinical samples and not an artifact of *in vitro* cell line propagation.

### G6PD overexpression is associated with increased PPP flux

**Figure 3:**
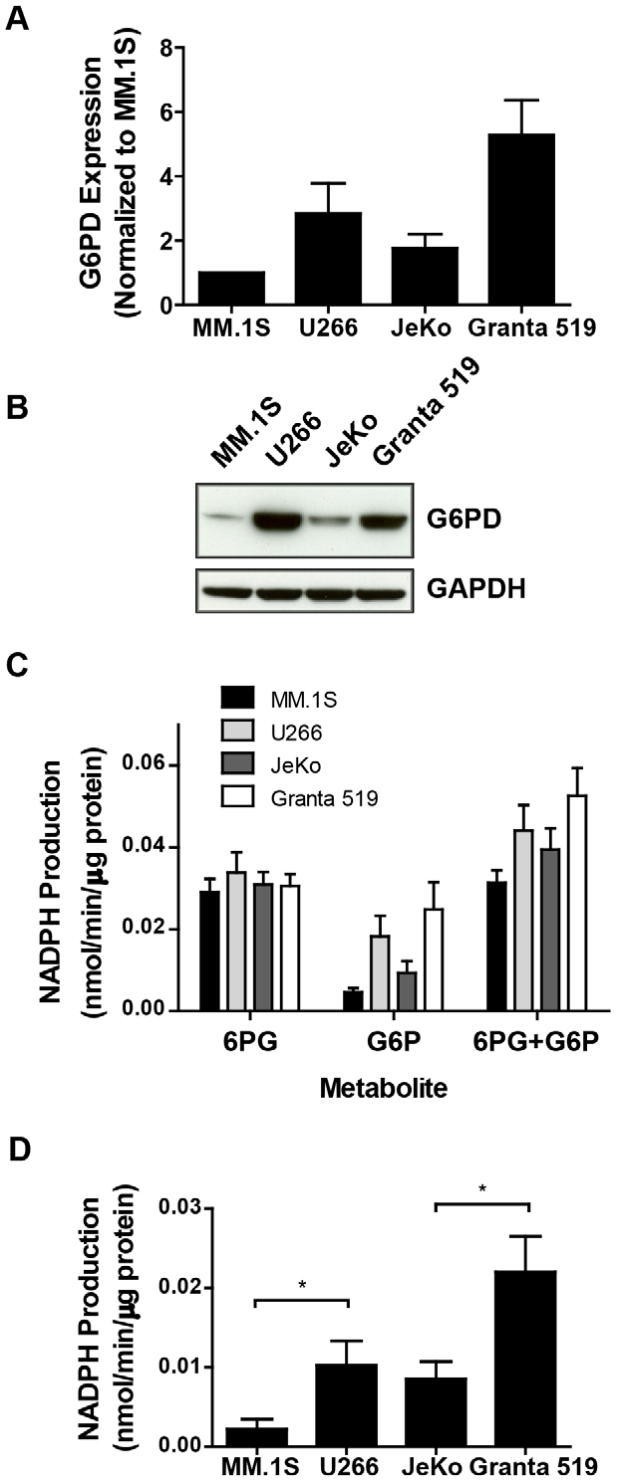
Elevated G6PD expression in resistant cell lines translates to greater enzymatic activity. (A) G6PD transcript abundance was evaluated through real time RT-PCR and normalized to the MM.1S cell line (n = 4). (B) G6PD protein abundance was evaluated through immunoblot analysis. GAPDH serves as a loading control. Representative blot from three independent experiments is shown. (C) Cell extracts were evaluated for 6PGD and G6PD activities via incubation with 6-phosphogluconate (6PG), glucose-6-phosphate (G6P) or both metabolites together. Activity was determined by measurement of NADP^+^ to NADPH conversion rates (n = 5 for MM cell lines, n = 4 for MCL cell lines). (D) G6PD-specific, 6PGD-independent activity was more stringently analyzed through subtraction of 6PG-induced NADPH generation from G6P/6PG-induced NADPH generation from data in part (C). Data in parts (A), (C), and (D) are means ± SEM. * P<.05 ** P<.01 *** P<.005.

We next sought to validate the microarray results pertaining to G6PD in the paired sensitive and resistant MM/MCL cell lines and establish a model system in which to interrogate the mechanistic impact of G6PD expression on 8-NH_2_-Ado/8-Cl-Ado activities through loss-of-function and gain-of-function studies. Quantitative real-time RT-PCR (Figure 3A) and immunoblot (Figure 3B) analyses confirmed increased G6PD expression in the resistant U266 and Granta 519 cell lines relative to the sensitive MM.1S and JeKo cells. These changes translated to increased enzymatic activity as determined by the rate of NADP^+^ to NADPH conversion in cell extracts (Figure 3C). Addition of NADP^+^ with either 6-phosphogluconate (6PG, the substrate for 6-phosphogluconate dehydrogenase) or glucose-6-phosphate (G6P, the substrate for G6PD) resulted in differential NADPH production between the representative cell lines only in the case of stimulation with glucose-6-phosphate. Furthermore, the absolute rate of NADPH production was markedly lower in the case of G6P supplementation. These data are consistent with a situation in which G6PD expression levels dictate flux through the pentose phosphate pathway by acting in a rate-determining fashion. To more stringently measure G6PD activity through exclusion of downstream 6-phosphogluconate dehydrogenase-dependent NADPH generation in G6P-treated cells, we subtracted the rate of NADPH production in extracts stimulated with 6PG from that in extracts stimulated with both 6PG and G6P. The results in Figure 3D demonstrate that G6PD protein abundance positively correlates with cellular activity of the enzyme.

### Elevated G6PD expression is necessary to maintain resistance to 8-NH_2_-Ado and 8-Cl-Ado

**Figure 4:**
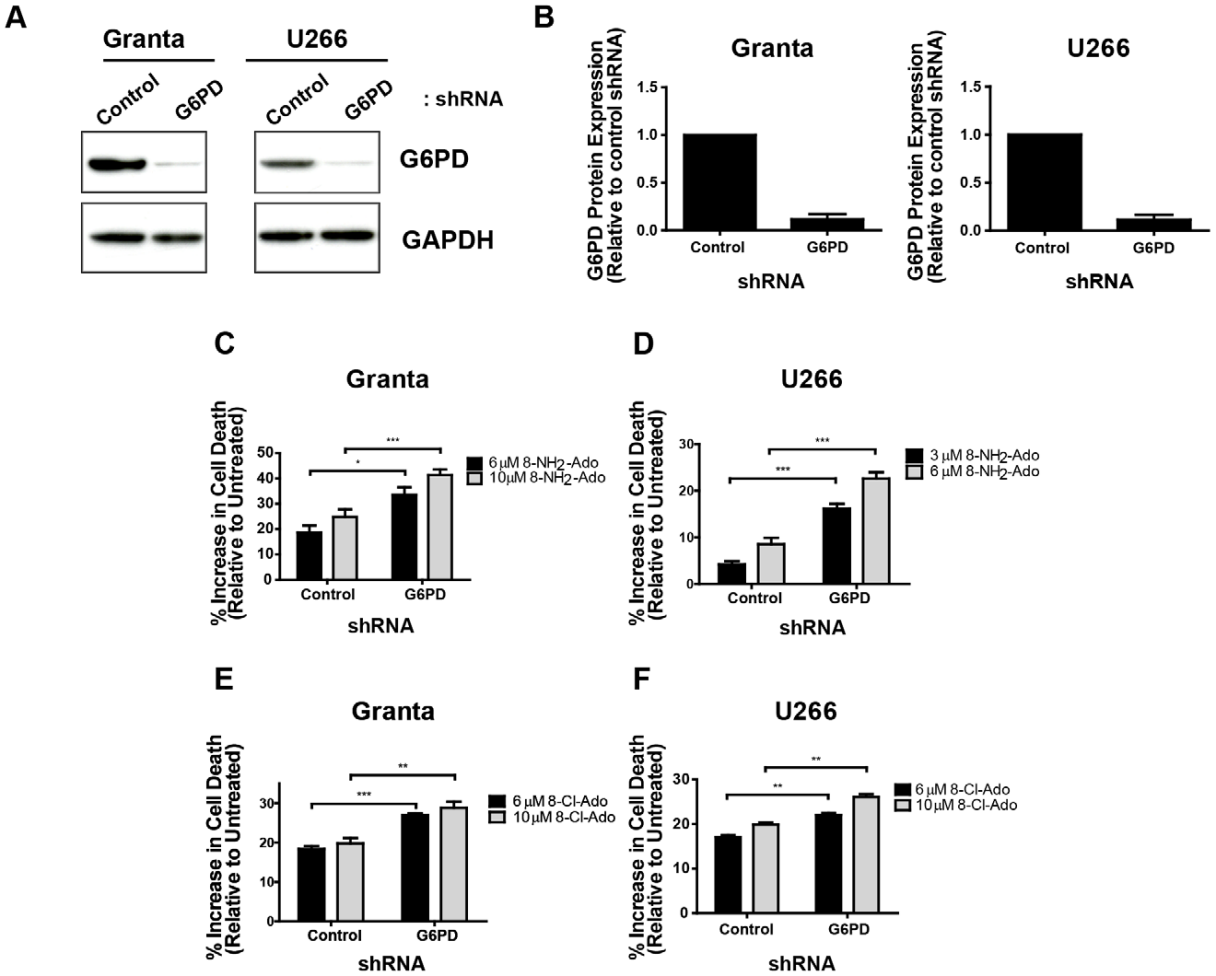
RNAi-mediated G6PD suppression sensitizes resistant cell lines to both 8-NH_2_-Ado and 8-Cl-Ado. (A) Resistant cell lines were transduced with lentiviral vectors encoding control, non-targeted or G6PD-targeted shRNAs. G6PD protein expression was analyzed by immunoblotting. Representative blots from three independent experiments are shown. (B) Densitometric quantification of G6PD expression from data in part (A) is shown and normalized to control shRNA-transduced cells. (C) Granta 519 cells were treated for 17 hours with the indicated concentrations of 8-NH_2_-Ado three days after transduction (n = 3). (D) U266 cell lines stably expressing distinct shRNAs were treated for 17 hours with the indicated concentrations of 8-NH_2_-Ado (n = 5). (E) Granta 519 cells were treated for 48 hours with the indicated concentrations of 8-Cl-Ado two days after transduction (n = 5). (F) U266 cell lines stably expressing distinct shRNAs were treated for 48 hours with the indicated concentrations of 8-Cl-Ado (n = 5). In parts (C)–(F), cell death was assessed solely by DAPI staining and data represent the increase in cell death relative to untreated cells (normalized to 0%). Data in parts (B)–(F) are means ± SEM. * P<.05 ** P<.01 *** P<.005.

Next, we addressed whether increased G6PD expression and activity observed in the U266 and Granta 519 cell lines is necessary to maintain resistance to 8-NH_2_-Ado and 8-Cl-Ado. To effectively reduce G6PD expression to levels similar to those in the sensitive cell lines, we transduced the resistant cell lines with control or G6PD-targeted shRNAs and evaluated protein abundance via immunoblot (Figure 4A). Densitometric quantification of immunoblot band intensities revealed approximately 90% suppression of G6PD expression in both cell lines (Figure 4B). Importantly, RNAi-mediated G6PD downregulation resulted in robust increases in 8-NH_2_-Ado sensitivity in both cell lines. Granta 519 cells displayed nearly a 2-fold increase in cell death upon G6PD knockdown at two concentrations of 8-NH_2_-Ado (Figure 4C). In the U266 cell line the effects were even more pronounced, with cell death tripling following 3 µM treatment and more than doubling following 6 µM treatment (Figure 4D). Representative flow cytometry dot plots of DAPI and AnnexinV staining in a single experiment depicted in Figure 4D are included as Figure S3. Both cell lines were also sensitized to 8-Cl-Ado treatment following G6PD reduction (Figure 4E–F), although the extent of chemosensitization was not nearly as substantial as observed in the context of 8-NH_2_-Ado. These studies confirm our hypothesis that G6PD overexpression is necessary to impart cellular resistance to 8-substituted adenosine analogues.

### Ectopic G6PD expression is insufficient to induce resistance to 8-NH_2_-Ado

**Figure 5:**
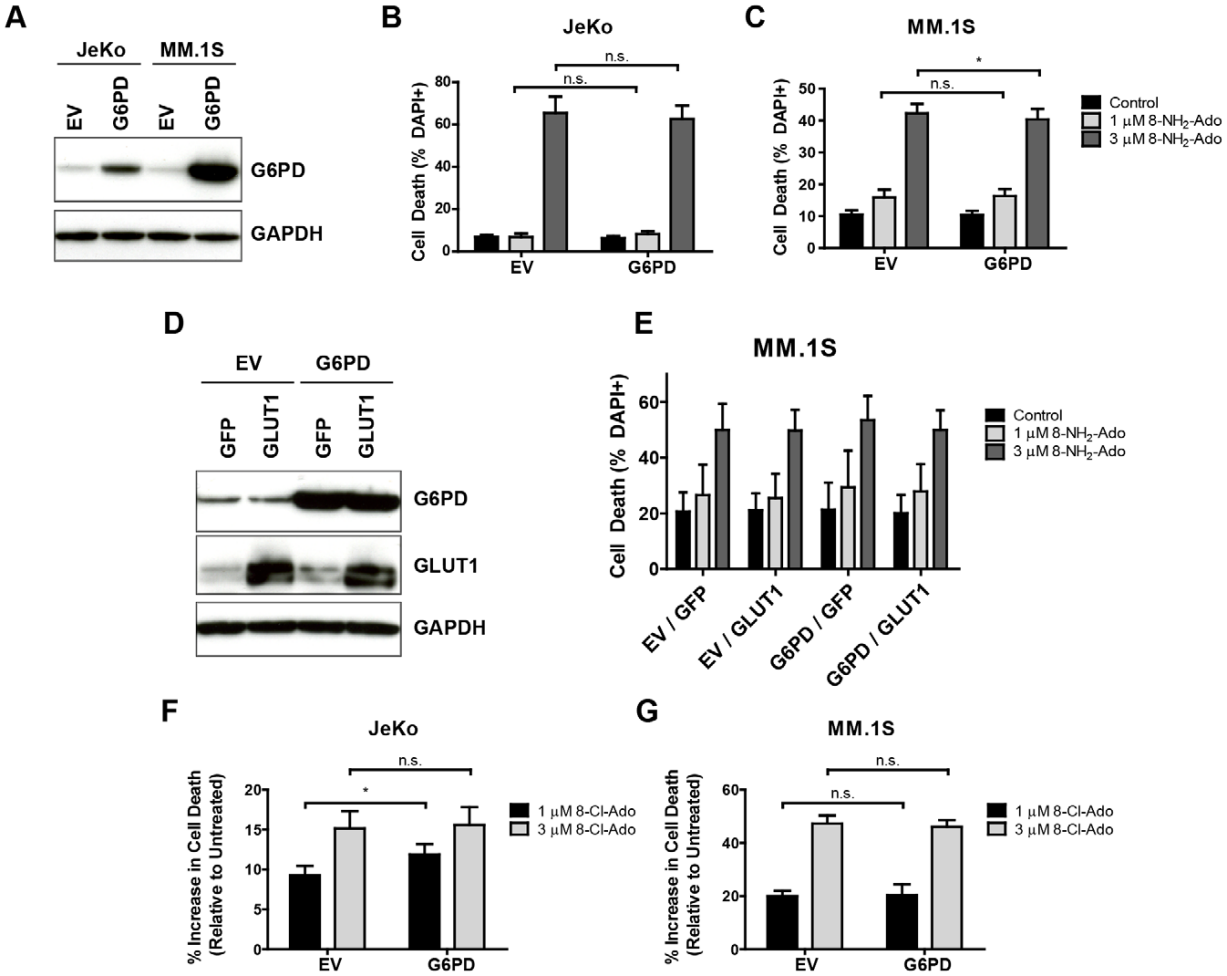
Ectopic expression of G6PD with and without GLUT1 is insufficient to abrogate 8-NH_2_-Ado sensitivity. (A) Sensitive cell lines were transduced with lentivirus harboring an empty vector control (EV) or G6PD cDNA (G6PD) and stable cell lines were generated. G6PD protein expression was analyzed by immunoblotting. A representative blot from three independent experiments is shown. (B) JeKo and (C) MM.1S stable cell lines were treated for 17 hours with the indicated concentrations of 8-NH_2_-Ado. Cell death was assessed solely by DAPI staining (n = 3 for JeKo, n = 5 for MM.1S). (D) MM.1S empty vector- and G6PD-expressing stable cell lines were transduced with GFP or GLUT1 cDNAs. G6PD and GLUT1 expression was analyzed by immunoblotting. A representative blot from three independent experiments is shown. (E) Cells from part (D) were treated for 17 hours with the indicated concentrations of 8-NH_2_-Ado. Cell death was assessed solely by DAPI staining (n = 3). (F) JeKo and (G) MM.1S stable cell lines were treated for 48 hours with the indicated concentrations of 8-Cl-Ado. In parts (F)–(G), cell death was assessed solely by DAPI staining (n = 5 for JeKo, n = 3 for MM.1S) and data represent the increase in cell death relative to untreated cells (normalized to 0%). Data in parts (B), (C), and (E)–(G) are means ± SEM. Statistically insignificant differences are indicated by n.s. except in part (E) in which all differences between cells receiving identical treatments were not statistically significant. * P<.05 ** P<.01 *** P<.005.

To more stringently evaluate this gene-drug association, we next asked whether ectopic G6PD expression is sufficient to abrogate the cytotoxicity of 8-NH_2_-Ado in the sensitive cell lines. We generated cell lines stably expressing an empty vector control (EV) or G6PD cDNA in the sensitive JeKo and MM.1S backgrounds via lentiviral transduction. As shown in Figure 5A, G6PD protein quantities were markedly increased in the G6PD-transduced lines, meeting or exceeding the endogenous expression levels of the resistant cell lines. We then tested whether this perturbation influenced 8-NH_2_-Ado activity by treating JeKo- (Figure 5B) and MM.1S-derived (Figure 5C) lines with 1 and 3 µM 8-NH_2_-Ado for 17 hours and measuring cell death induction. Representative flow cytometry dot plots of DAPI and AnnexinV staining in a single experiment depicted in Figure 5C are included as Figure S4. While there was a slight reduction in cytotoxicity which reached statistical significance in the G6PD-overexpressing MM.1S cells treated with 3 µM 8-NH_2_-Ado, no cytoprotection was afforded in any other instance. These observations lead us to conclude that enforced increases in G6PD expression alone are insufficient to blunt 8-NH_2_-Ado cytotoxicity. In a recent publication, we demonstrated that 8-NH_2_-Ado acutely reduces the rate of glucose consumption in MM.1S cells which is associated with redistribution of the glucose transporter GLUT4 from a plasma membrane/cytosolic localization pattern to an aggregation within the *trans*-Golgi network [10]. Given the fact that no short-term effects were noted on GLUT1 expression or subcellular localization, we successfully rescued the decrease in glucose uptake induced by 8-NH_2_-Ado exposure through GLUT1 overexpression. To ensure that glucose transport inhibition was not overriding the impact of G6PD overexpression and thus confounding interpretation of the results, we overexpressed G6PD and GLUT1 simultaneously to maintain glucose metabolism during treatment. The stable MM.1S cell lines represented in Figure 5A were transduced with GFP and GLUT1 cDNAs and modulation of G6PD/GLUT1 expression was assessed by immunoblot analysis (Figure 5D). As depicted in Figure 5E, G6PD overexpression with and without concomitant GLUT1 overexpression was not sufficient to alter 8-NH_2_-Ado sensitivity. In agreement with the results pertaining to 8-NH_2_-Ado treatment, G6PD overexpression also failed to induce resistance to 8-Cl-Ado in both JeKo and MM.1S cells (Figure 5F–G). Taken together, our data define a functional, requisite role for increased G6PD expression in maintaining resistance to 8-NH_2_-Ado/8-Cl-Ado but suggest that co-regulation of an unidentified cellular process in addition to elevated PPP flux is necessary to induce *de novo* drug resistance in MM and MCL cells.

## Discussion

MM and MCL cell lines display substantial heterogeneity with respect to the relationship between intracellular 8-NH_2_-ATP/8-Cl-ATP accumulation and sensitivity to the respective prodrugs. Therefore, we were interested in identifying gene expression patterns predictive of cellular responsiveness to these agents. To address this issue, we implemented a novel integrative chemoinformatic screen to uncover gene-drug associations applicable to both 8-NH_2_-Ado and 8-Cl-Ado with particular relevance in the context of B cell malignancies. This experimental approach was designed to capitalize on the large sample size and associated statistical power inherent in the primary screen of the NCI-60 cell line panel. The secondary screen of paired MM and MCL cell lines employed a relatively tolerant cutoff for the identification of differentially expressed genes (>1.5 fold change) given that it essentially served as a filtering mechanism; the resultant dataset was subsequently integrated with the dataset derived from the primary screen to select only overlapping genes. Through this stringent analysis, we identified 7 genes which are associated with responsiveness to both 8-NH_2_-Ado and 8-Cl-Ado. An association between elevated *G6PD* expression and chemoresistance was uncovered from this gene signature and, through a series of functional assays, we demonstrate herein that high G6PD expression is necessary but not sufficient to induce resistance to 8-NH_2_-Ado and 8-Cl-Ado. This finding corroborates and expands upon our previous findings that glucose deprivation is capable of sensitizing cells to 8-NH_2_-Ado but interventions designed to maintain glucose metabolism during 8-NH_2_-Ado treatment fail to induce resistance. One caveat regarding the insufficiency of ectopic G6PD expression (with or without GLUT1) to induce resistance to 8-NH_2_-Ado entails the potential for inactivation of the enzyme to occur in the absence of growth factor receptor signaling [33], [34]. Importantly, the inability of G6PD overexpression to alleviate sensitivity to this agent may be predicated upon the widespread deactivation of growth-related intracellular signaling pathways by 8-NH_2_-Ado [3], [35], resulting in the sequestration and inhibition of G6PD. In any event, our current results highlight the oxidative PPP as the critical metabolic pathway downstream of glucose transport which is capable of modulating the activities of 8-NH_2_-Ado and 8-Cl-Ado. These results represent an important addition to the increasing appreciation of the widespread importance of *G6PD* to the autonomous resistance of tumor cells to anticancer therapies.

The G6PD enzyme regulates synthesis of two intracellular metabolites to ultimately determine the efficacy of certain chemotherapeutics. The first of these metabolites, NADPH, is directly produced by G6PD during the oxidation of glucose-6-phosphate and indirectly produced by stimulating the downstream PPP enzyme 6-phosphogluconate dehydrogenase. The positioning of G6PD as the rate-determining enzyme in the PPP renders this enzyme a primary source of NADPH for many tissues. One of the most prominent NADPH-dependent cellular functions consists of the reduction of one molecule of glutathione disulfide to form two molecules of glutathione via glutathione reductase activity. NADPH serves as the reducing equivalent required to drive the chemical reaction forward, ultimately counteracting oxidation of the glutathione pool within cells. Glutathione, in turn, is a substrate for numerous enzymes required to detoxify reactive oxygen species (ROS) capable of eliciting cellular damage and apoptosis via direct biomolecular oxidation (including amino acid residues and DNA bases). The antitumor activity of many classical chemotherapeutics can be partially ascribed to the induction of oxidative stress. For example, paclitaxel, an inhibitor of microtubule dynamics and mitosis, induces a marked increase in catalase-inhibitable oxidation of the redox-sensitive probe H_2_DCFDA in breast cancer cell lines [36]. Importantly, paclitaxel-resistant cell lines derived from the parental squamous lung carcinoma line SKMES-1 exhibit upregulation of the PPP enzymes G6PD and 6-phosphogluconolactonase in addition to various glutathione-dependent enzymes (i.e. peroxiredoxins, protein disulfide isomerase) capable of combating oxidative stress [37]. These observations point to maintenance of redox homeostasis as a primary mechanism leading to paclitaxel resistance and suggest that the association between elevated G6PD expression and resistance to 8-NH_2_-Ado and 8-Cl-Ado may also occur through a glutathione-dependent process. Interestingly, 8-Cl-ATP has been predicted through computational docking studies to interact with ATP synthase in a manner similar to the non-hydrolyzable complex V inhibitor ANP [38]. Given the fact that oligomycin-mediated complex V inhibition results in increased hydrogen peroxide production [39], the putative binding of 8-Cl-ATP to ATP synthase may elicit a similar increase in ROS and lead to oxidative stress-induced apoptosis.

Alternatively, G6PD-dependent NADPH production could promote the glutathionylation and detoxification of 8-NH_2_-Ado and 8-Cl-Ado or their phosphorylated derivatives and thereby influence cell fate decisions following treatment. Evidence for the direct chemical conjugation of glutathione with cyclophosphamide has been provided through mass spectrometry analysis [40] and has been postulated to account for glutathione depletion-mediated cyclophosphamide sensitization in the K562 leukemia cell line [41]. However, this mechanism seems unlikely to be relevant with respect to 8-Cl-Ado as HPLC/UV analysis of radio-labeled metabolites failed to identify peaks corresponding to glutathionylated drug species in cell extracts [42].

In direct opposition to the first mechanism presented, NADPH can also serve to increase ROS production through the NADPH oxidase (NOX) family of enzymes. These heteromeric, membrane-bound protein complexes produce superoxide anions by transferring electrons from NADPH to molecular oxygen. In cells with low endogenous ROS levels, superoxide production via NOX activity can stimulate growth-supporting, anti-apoptotic signaling pathways through oxidation of critical cysteine residues within the active sites of protein tyrosine phosphatases [43], [44] or cause global changes in gene transcription programs through HIF-1α stabilization [45]. Precise evaluation of the changes in ROS levels following treatment of resistant and sensitive cell lines appears to be a promising avenue for further investigation.

The second metabolite principally controlled by G6PD activity is ribose-5-phosphate, a precursor for *de novo* purine synthesis. One hypothetical connection between G6PD activity and sensitivity to 8-NH_2_-Ado and 8-Cl-Ado lies in the competitive interaction of endogenous adenosine molecules with 8-substituted adenosine analogues for access to enzymes responsible for nucleoside/nucleotide phosphorylation, such as adenosine kinase and ATP synthase. Cells exhibiting greater capacity for ribose-5-phosphate generation and adenosine synthesis could potentially suppress accumulation of 8-NH_2_-ATP and 8-Cl-ATP and thus limit exposure of intracellular targets to these active metabolites. This explanation, however, seems highly unlikely based on two observations. First, as mentioned throughout this article, resistant cell lines do not display reduced intracellular levels of triphosphorylated analogues relative to sensitive lines. Second, reduced oxygen tension is sufficient to restore clonogenic survival to G6PD-null murine cells [46], suggesting that glutathione-dependent protection from oxidative stress is a crucial effect of G6PD activity whereas ribose-5-phosphate synthesis is dispensable. This concept is supported by the fact that ribose-5-phosphate can be synthesized from glycolytic intermediates via an alternative metabolic pathway constituted by transaldolase and transketolase.

Our insight into the association between elevated G6PD expression and resistance to RNA-directed nucleoside analogues strengthens the ties between this metabolic enzyme and the chemoresistant phenotype in cancer and provides valuable information for future studies. Specifically, further examination of this gene-drug association in clinical trials of 8-Cl-Ado and 8-NH_2_-Ado will be of great importance. Additionally, investigations of potential synergistic interactions between agents targeting G6PD, the PPP or downstream processes including glutathione generation (e.g. buthionine S′R′-sulfoximine) and 8-NH_2_-Ado/8-Cl-Ado are warranted. Elucidation of unique aspects of the mechanisms of action of these novel agents and exploration of the molecular networks regulating their activities will facilitate optimal clinical application of these promising drugs and ultimately lead to maximal benefit for patients.

## Materials and Methods

### Cell culture

The MM.1S cell line was developed in our laboratory [47]. The U266 and JeKo cell lines were obtained from ATCC. Granta 519 cells [48] were kindly provided by Dr. Varsha Gandhi. MM.1S, U266, and JeKo cells were cultured in complete RPMI 1640 (GIBCO) supplemented with 10% fetal bovine serum (FBS), 2 mM glutamine, 100 U/mL penicillin, 100 mg/ml streptomycin, 2.5 µg/ml fungizone and 0.5 µg/ml plasmocin (InvivoGen) and maintained in a 37°C incubator with 5% CO_2_. Granta 519 cells were cultured similarly except that the base medium utilized was DMEM.

### Chemicals and reagents

Standard chemicals, including G418 and puromycin, were purchased from Sigma Aldrich. 8-amino-adenosine was purchased from RI chemicals. 8-chloro-adenosine was synthesized through the Rapid Access to Intervention Development (RAID) program (Developmental Therapuetics Program, National Cancer Institute). Antibodies to GLUT1 and G6PD were purchased from Abcam. Antibody to GAPDH was from Millipore. AnnexinV-fluorescein isothiocyanate (FITC) and AnnexinV-allophycocyanin (APC) were purchased from BD Biosciences. Real time PCR Taqman gene expression assays were purchased from Applied Biosystems (G6PD, Assay ID Hs00166169_m1) or PrimerDesign (YWHAZ, RPL13A, EIF4A2). NADP^+^, NADPH, 6-phosphogluconate, and glucose-6-phosphate were purchased from Sigma Aldrich. Control and G6PD shRNAs in the lentiviral vector pLKO.1 were purchased from Sigma Aldrich. G6PD cDNA (I.M.A.G.E. clone ID 2822640) in the vector pOTB7 was purchased from ATCC. The lentiviral vectors used for cDNA overexpression include: pLVX-IRES-Neo (Clontech) and pReceiver-Lv-151 (Genecopoeia, harboring GFP and GLUT1 cDNAs).

### Cell viability/death assays

DAPI staining with AnnexinV-FITC was utilized to assess cell death via flow cytometry in Figure 1A. In all other cell death assays, DAPI staining was used alone without AnnexinV. A Dako CyAn™ ADP analyzer was used for data collection and FCS Express V3 software was used for data analysis (De Novo Software). For all cell death experiments, cells were plated in 6 well plates at densities of .125*10^6^–.25*10^6^ cells/mL. 8-NH_2_-Ado treatments lasted 17 hours while 8-Cl-Ado treatments lasted 48 hours. For cell death experiments in which baseline viability was impacted by either G6PD silencing or overexpression, the fraction of DAPI-negative cells in each sample was first divided by the fraction of DAPI-negative cells in the corresponding untreated sample exhibiting the same genetic manipulation. This normalized ratio was then multiplied by 100 to convert this fraction to a percentage. Finally, these values were subtracted from 100% to arrive at a normalized percentage of cell death in each sample such that untreated samples exhibited normalized cell death values of 0%. This normalization method enabled direct comparisons of drug activity across distinct genetic subgroups by excluding the impact of G6PD overexpression/suppression on basal cell viability. For cell death experiments in which baseline viability was not impacted by the genetic perturbation of interest, normalization was not required and raw percentages of DAPI-positive cells were used for analysis of drug sensitivity.

### Gene expression microarray data collection and analysis

Publicly available gene expression data pertaining to the NCI-60 cell line panel were obtained from http://www.genome.wi.mit.edu/mpr/nci60/nci60.html. Experimental protocols for data collection and processing are described by Staunton et al [49]. The dataset used was collected on the Affymetrix HuGeneFL chip. For microarrays of the paired sensitive and resistant myeloma and mantle cell lymphoma cell lines, RNA expression analysis was performed using the Illumina Human-HT12 BeadChip which provides coverage of around 48,800 genes and expressed sequence tags. Total RNA was extracted from MM.1S, JeKo, U266 and Granta 519 cells and the poly(A)^+^ fraction amplified and labeled by with the TargetAmp 1-Round Aminoallyl-aRNA Amplification Kit (Epicentre Biotechnologies). Labeled RNA was hybridized to microarrays and raw signal intensities of each probe were obtained using Illumina Beadstudio data analysis software and imported to the Lumi package of Bioconductor for data transformation and normalization [50]–[52]. A/P call detection was performed based on detection *P* value. 21,629 out of 48,802 probes with *P* less than 0.01 were considered as valid signals. Differentially expressed genes were identified using an Analysis of Variance (ANOVA) model with empirical Bayesian variance estimation [53]. The problem of multiple comparisons was corrected using the false discovery rate (FDR). Initially, genes were identified as being differentially expressed on the basis of a statistically significant (raw *P*-value<0.01 and fdr <0.05 and 1.5-fold difference) in expression level in different samples. All microarray data collection and analysis was performed in compliance with MIAME guidelines. Data have been deposited in NCBI's Gene Expression Omnibus and are accessible through GEO Series accession number GSE38145 (GSM935812–GSM935856).

### Determination of GI_50_ values for 8-Cl-Ado and assessment of gene-drug associations

The 60 cell lines comprising the NCI-60 panel were assayed for sensitivity to 8-Cl-Ado as part of the Rapid Access to Intervention Development (RAID) initiative offered through the Developmental Therapuetics Program at the National Cancer Institute [54] (see: http://dctd.cancer.gov/ProgramPages/dtp/major_drug_development.htm). Cells were treated with 8-Cl-Ado for 48 hours and growth inhibition was determined by sulforhodamine B assay. The drug concentration eliciting 50% growth inhibition in each cell line was provided as the GI_50_ and subsequently log_10_ transformed to facilitate comparisons between cell lines. log_10_(GI_50_) values were compared with expression values obtained from 7,129 unique probes across all cell lines and linear regression analysis was performed to calculate Pearson correlation coefficients for gene-drug pairs.

### Gene expression studies in primary cells utilizing microarray data from online repositories

Oncomine™ (Compendia Bioscience, Ann Arbor, MI) was used for analysis and visualization of gene expression data in MM, CLL, and MCL primary samples and corresponding normal tissues.

### RNA extraction and real-time RT-PCR

RNA isolation was performed using the RNeasy Mini Kit (Qiagen). Reverse transcription and real time PCR were carried out according to standard protocols. Data was collected on an ABI 7900 HT instrument. Normalization for differential loading was achieved through the geNorm method [55] by generating a normalization factor from the expression of three endogenous control genes (YWHAZ, RPL13A, and EIF4A2).

### Immunoblot analysis

Whole cell lysates were prepared with the Complete Lysis-M buffer (Roche Applied Science) supplemented with phosphatase inhibitor cocktail tablets (Roche). Immunoblotting was carried out according to a standard protocol with species-specific, horseradish peroxidase-linked secondary antibodies (Cell Signaling Technology). Where applicable, immunoblots were scanned and band intensities quantified using ImageJ software.

### G6PD activity assay

Cells were harvested and resuspended in PBS. Cellular homogenization was carried out using a Qiagen TissueLyser LT (50 Hz for 5 min), after which the lysates were centrifuged and the supernatant collected. Protein concentrations were determined and samples diluted in PBS to 0.5 µg/µL. G6PD and 6PGD enzyme activities were then measured via monitoring of NADP^+^ to NADPH conversion. 50 µL of each cell sample was added to 50 µL of buffer (50 mM Tris-EDTA, 1 mM MgCl, pH 8.1) [34] with reaction substrates added (200 µM G6P or 6PG with 200 µM NADP^+^). To determine the activity of both enzymes simultaneously, the substrate mixture consisted of 200 µM G6P, 200 µM 6PG, and 400 µM NADP^+^. Enzyme activity was determined spectrophotometrically by a kinetic assay measuring fluorescence at 341 nm due to the reduction of NADP^+^ to NADPH. Purified G6PD enzyme was incubated with reaction mixtures as a control. Data was gathered for 30 minutes at 1 minute intervals. To ensure that G6PD activity measurements were not confounded by induction of downstream 6PGD activity, a more stringent analysis was performed by calculating the difference between total enzyme activity (G6PD and 6PGD) and 6PGD activity alone [56].

### Cloning

The G6PD open reading frame (ORF) was PCR-amplified from a construct purchased from ATCC using primers designed to append a 5′ EcoRI restriction site and a 3′ SpeI site. Both the PCR product and the bicistronic lentiviral vector pLVX-IRES-Neo were double digested with EcoRI and SpeI and purified accordingly. The G6PD ORF was ligated directionally into the vector overnight and the product was used to transform bacteria. Selected colonies were screened for the presence of the ligation product and DNA sequencing verified the presence and sequence of the inserted G6PD ORF.

### RNA interference

The G6PD-targeted shRNA used (ID no. TRCN0000025817) was in the lentiviral vector pLKO.1 and produced the mature antisense sequence 5′-TTCACGTTCTTGTATCTGTTG-3′. Granta 519 cells were transduced prior to each experiment due to the delayed development of significant cytotoxicity associated with G6PD suppression. 8-NH_2_-Ado treatments were started three days after transduction and lasted 17 hours while 8-Cl-Ado treatments began two days after transduction and lasted 48 hours. G6PD expression was determined three days post-transduction. Puromycin was not added to Granta 519 cultures since stable cell lines were not able to be generated from G6PD-silenced cells and the RNAi experiments were of relatively short duration. U266 cells were transduced and selected in 0.25 µg/mL puromycin for seven days to generate stable cell lines. These lines were used for experiments involving 8-NH_2_-Ado/8-Cl-Ado treatment within one month of selection. Immunoblot analyses confirmed that G6PD suppression was successfully maintained over this timeframe. Similarly to Granta 519 cells, 8-NH_2_-Ado treatments lasted 17 hours and 8-Cl-Ado treatments lasted 48 hours for U266 cells.

### Lentiviral production and cell line transduction

Large-scale production of high-titer lentiviral vectors was carried out according to an established protocol [57]. Briefly, 14 cm dishes of log-phase HEK293T cells (purchased from Clontech) were transfected via the CaCl_2_/HBS method. Virus was collected in UltraCulture serum-free medium (Lonza) and cold-precipitated in polyethylene glycol via centrifugation. Viral pellets were resuspended in fixed volumes of PBS and stored in single-use aliquots at −80°C until transduction. 10^6^ to 2*10^6^ cells were transduced per well in 6 well plates. Cells were plated at a volume of 1.5 ml per well in UltraCulture serum-free medium containing 2.5 µg/ml polybrene (Sigma). 0.5% FBS was added for MM.1S cells due to their hypersensitivity to serum deprivation. Viral aliquots were removed from the −80°C freezer and allowed to thaw slowly at RT. After virus addition, cells were centrifuged at 2500 RPM at RT for 1.5 hours. After centrifugation, cells were placed into a 37°C incubator and incubated another 1.5–2 hours with the exception of MM.1S cells, which were harvested immediately to preserve viability. Cells were washed 1× with PBS to remove virus and resuspended in fresh medium. After 2 days, 750 or 850 µg/mL G418 was added to MM.1S and JeKo cultures, respectively, for seven days to select for successfully transduced cells and generate stable cell lines. Likewise, 0.25 µg/mL puromycin was added to U266 cultures to generate stable cell lines.

### Statistical analysis

All data presented are derived from at least 3 independent experiments. Two-tailed *P* values were calculated using paired (for *in vitro* cell line experiments) or unpaired (for primary cell microarray studies) t tests with GraphPad Prism software. *P* values<0.05 were considered to be statistically significant. Correlations between gene expression profiles and GI_50_ values for 8-amino-adenosine or 8-chloro-adenosine were evaluated first by linear regression and calculation of the Pearson correlation coefficient. The *t*-statistic was calculated from r based on the formula: 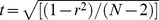.

Finally, the *t*-statistic and number of degrees of freedom were utilized to generate a two-tailed *P* value for each probe representing the statistical significance of the gene-drug association.

## Supporting Information

Figure S1 **Scatter plot representation of linear association between 8-Cl-Ado GI_50_ values and** ***LGALS1*** **expression.** Normalized gene expression values pertaining to *LGALS1* (probe set ID J04456_at) are graphed on the x-axis while corresponding log_10_(GI_50_) values for each cell line are plotted on the y-axis. Data points for each cell line are replaced with the cell line name. *LGALS1* is the most highly associated gene with resistance to 8-Cl-Ado (r = 0.5418).
(TIFF)

Figure S2 **Real-time RT-PCR validation of differential gene expression patterns determined through microarray analysis.** (A) Transcript abundances for the gene products of *MYC, VIM*, *ACTN1*, *THY1*, and *LGALS1* were measured in the paired MM and MCL cell lines by real-time RT-PCR and normalized to the indicated cell line. (B) Cyclin D1 transcript abundance was measured by real-time RT-PCR in the paired MM and MCL cell lines and normalized to the indicated cell line. Three distinct primer/probe sets detecting the cyclin D1 transcript were used to confirm specificity in the MCL lines. The transcript regions targeted by the primer/probe sets are as follows: 1 spans the exon 3–4 boundary, 2 spans the exon 4–5 boundary, and 3 spans the exon 2–3 boundary. (C) Cyclin D1 protein abundance was evaluated through immunoblot analysis. GAPDH serves as a loading control. Representative blot from three independent experiments is shown. Densitometric quantification of band intensities from the three separate blots was also performed and values are normalized to the U266 cell line. Data in parts (A)–(C) are means ± SEM (n = 3).
(TIF)

Figure S3 **Representative flow cytometry dot plots demonstrate an increase in DAPI staining upon 8-NH_2_-Ado treatment in U266 cells.** Raw data from a single experiment represented by Figure 4D is included to demonstrate DAPI positivity of 8-NH_2_-Ado-treated U266 cells expressing either control or G6PD-targeted shRNA. (A) DAPI and (B) AnnexinV-FITC/DAPI dot plots are displayed.
(TIF)

Figure S4 **Representative flow cytometry dot plots demonstrate an increase in DAPI staining upon 8-NH_2_-Ado treatment in MM.1S cells.** Raw data from a single experiment represented by Figure 5C is included to demonstrate DAPI positivity of 8-NH_2_-Ado-treated MM.1S cells expressing either an empty vector control or G6PD cDNA. (A) DAPI and (B) AnnexinV-FITC/DAPI dot plots are displayed.
(TIF)

Table S1 **All gene expression patterns positively and significantly correlated with 8-Cl-Ado resistance.** Genes exhibiting a positive correlation between expression level and 8-Cl-Ado resistance in the NCI-60 cell line panel (P<.05) are listed.
(XLSX)

Table S2 **All gene expression patterns negatively and significantly correlated with 8-Cl-Ado resistance.** Genes exhibiting a negative correlation between expression level and 8-Cl-Ado resistance in the NCI-60 cell line panel (P<.05) are listed.
(XLSX)
