# Supplementary figures and images for: Integrative Gene Expression Profiling Reveals G6PD-Mediated Resistance to RNA-Directed Nucleoside Analogues in B-Cell Neoplasms
Source: PLoS One. 2012 Jul 27;7(7):e41455. doi: 10.1371/journal.pone.0041455 (PMC3407247; doi:10.1371/journal.pone.0041455)

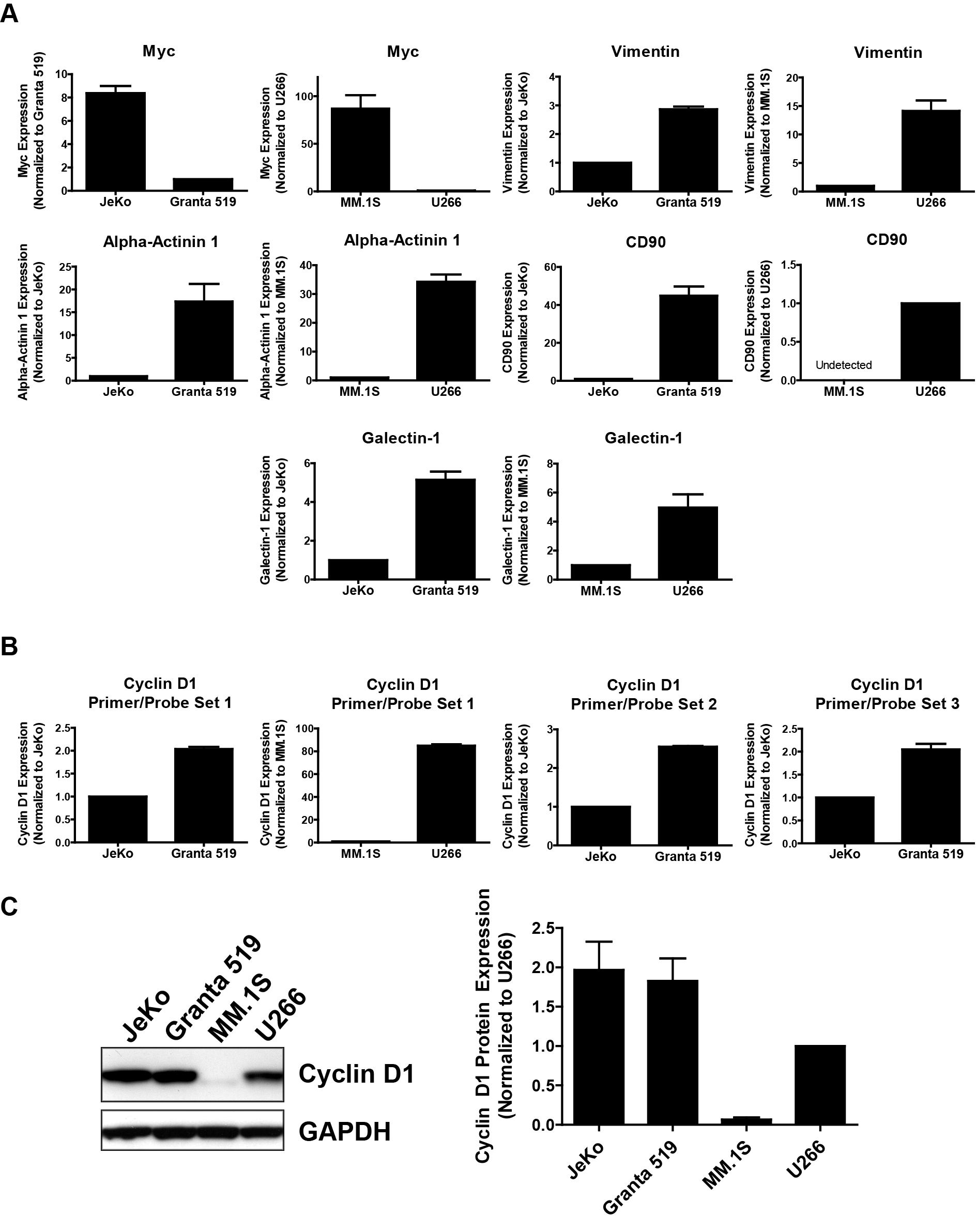

Supplement: Figure S2 — Real-time RT-PCR validation of differential gene expression patterns determined through microarray analysis. (A) Transcript abundances for the gene products of MYC, VIM, ACTN1, THY1, and LGALS1 were measured in the paired MM and MCL cell lines by real-time RT-PCR and normalized to the indicated cell line. (B) Cyclin D1 transcript abundance was measured by real-time RT-PCR in the paired MM and MCL cell lines and normalized to the indicated cell line. Three distinct primer/probe sets detecting the cyclin D1 transcript were used to confirm specificity in the MCL lines. The transcript regions targeted by the primer/probe sets are as follows: 1 spans the exon 3–4 boundary, 2 spans the exon 4–5 boundary, and 3 spans the exon 2–3 boundary. (C) Cyclin D1 protein abundance was evaluated through immunoblot analysis. GAPDH serves as a loading control. Representative blot from three independent experiments is shown. Densitometric quantification of band intensities from the three separate blots was also performed and values are normalized to the U266 cell line. Data in parts (A)–(C) are means ± SEM (n = 3). (TIF) [file pone.0041455.s002.tif]

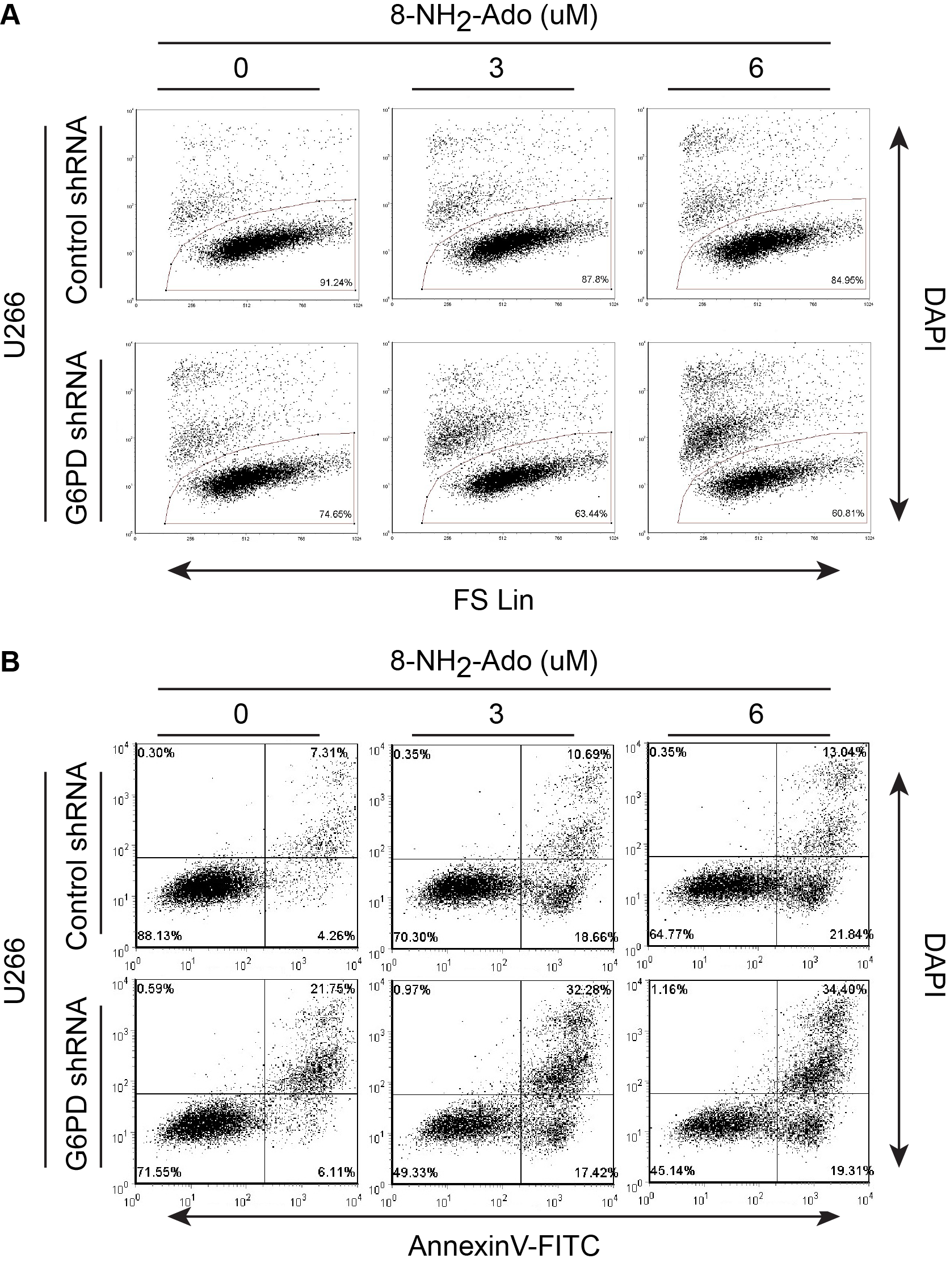

Supplement: Figure S3 — Representative flow cytometry dot plots demonstrate an increase in DAPI staining upon 8-NH2-Ado treatment in U266 cells. Raw data from a single experiment represented by Figure 4D is included to demonstrate DAPI positivity of 8-NH2-Ado-treated U266 cells expressing either control or G6PD-targeted shRNA. (A) DAPI and (B) AnnexinV-FITC/DAPI dot plots are displayed. (TIF) [file pone.0041455.s003.tif]

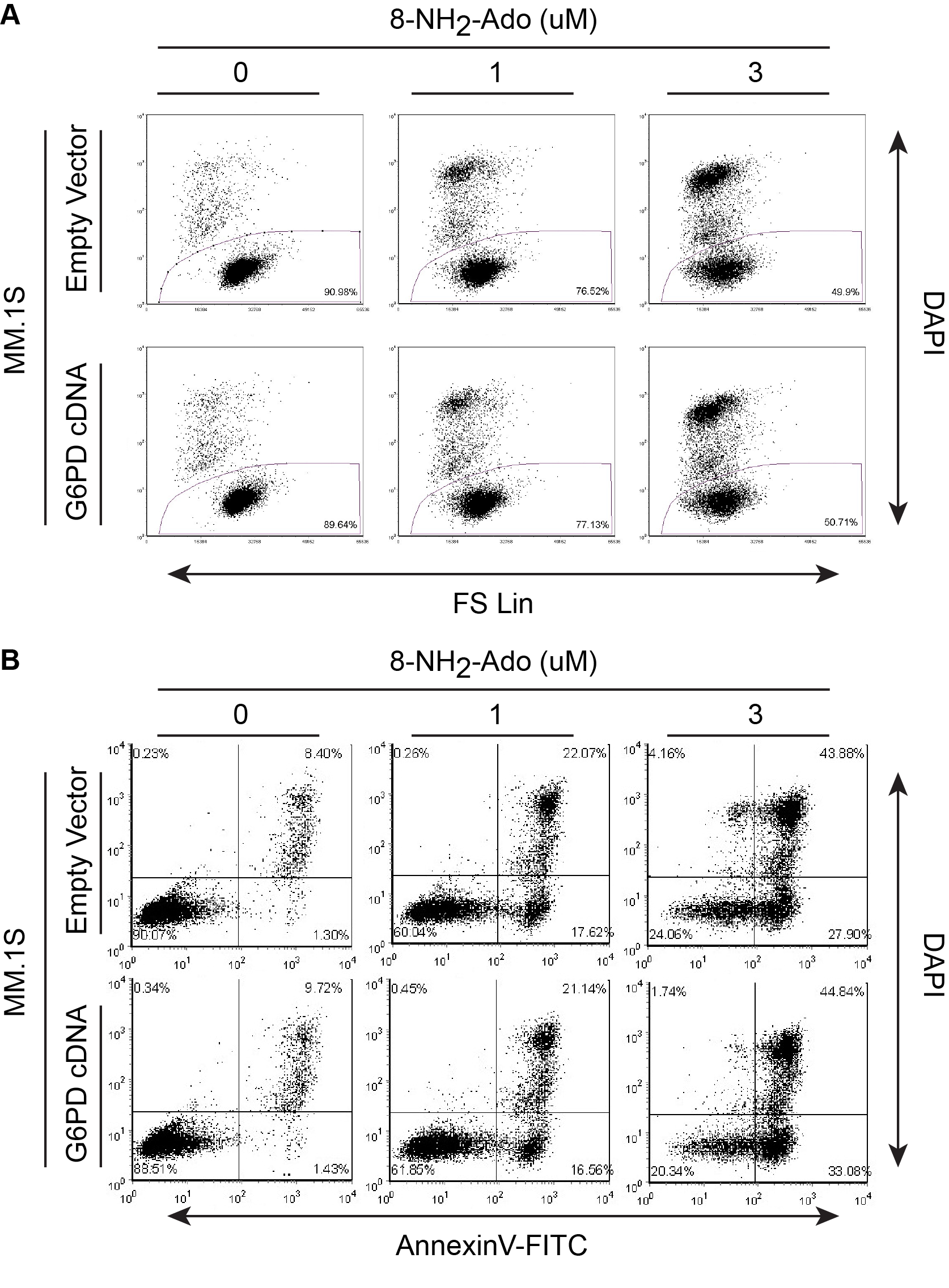

Supplement: Figure S4 — Representative flow cytometry dot plots demonstrate an increase in DAPI staining upon 8-NH2-Ado treatment in MM.1S cells. Raw data from a single experiment represented by Figure 5C is included to demonstrate DAPI positivity of 8-NH2-Ado-treated MM.1S cells expressing either an empty vector control or G6PD cDNA. (A) DAPI and (B) AnnexinV-FITC/DAPI dot plots are displayed. (TIF) [file pone.0041455.s004.tif]
